# RRM2 Mediates the Anti-Tumor Effect of the Natural Product Pectolinarigenin on Glioblastoma Through Promoting CDK1 Protein Degradation by Increasing Autophagic Flux

**DOI:** 10.3389/fonc.2022.887294

**Published:** 2022-05-11

**Authors:** Haiping Jiang, Dongzhi Zhang, Karpov Denis Aleksandrovich, Junyi Ye, Lixiang Wang, Xiaofeng Chen, Ming Gao, Xinzhuang Wang, Tao Yan, He Yang, Enzhou Lu, Wenwu Liu, Cheng Zhang, Jianing Wu, Penglei Yao, Zhenying Sun, Xuan Rong, Sokhatskii Andrei Timofeevich, Safin Shamil Mahmutovich, Zhixing Zheng, Xin Chen, Shiguang Zhao

**Affiliations:** ^1^ Department of Neurosurgery, The First Affiliated Hospital of Harbin Medical University, Harbin, China; ^2^ Department of Neurosurgery, Key Colleges and Universities Laboratory of Neurosurgery in Heilongjiang Province, Harbin, China; ^3^ Institute of Neuroscience, Sino-Russian Medical Research Center, Harbin Medical University, Harbin, China; ^4^ Department of Neurosurgery, The Affiliated Cancer Hospital of Harbin Medical University, Harbin, China; ^5^ Department of Neurosurgery and Medical Rehabilitation, Bashkir State Medical University, Ufa, Russia; ^6^ Department of Undergraduate, Suffolk University, Boston, MA, United States; ^7^ Department of Neurosurgery, Shenzhen University General Hospital, Shenzhen, China

**Keywords:** pectolinarigenin, glioblastoma, RRM2, CDK1, autophagic flux

## Abstract

The natural product pectolinarigenin exerts anti-inflammatory activity and anti-tumor effects, and exhibits different biological functions, particularly in autophagy and cell cycle regulation. However, the antineoplastic effect of pectolinarigenin on glioblastoma (GBM) remains unclear. In the present study, we found that pectolinarigenin inhibits glioblastoma proliferation, increases autophagic flux, and induces cell cycle arrest by inhibiting ribonucleotide reductase subunit M2 (RRM2), which can be reversed by RRM2 overexpression plasmid. Additionally, pectolinarigenin promoted RRM2 protein degradation *via* autolysosome-dependent pathway by increasing autophagic flow. RRM2 knockdown promoted the degradation of CDK1 protein through autolysosome-dependent pathway by increasing autophagic flow, thereby inhibiting the proliferation of glioblastoma by inducing G2/M phase cell cycle arrest. Clinical data analysis revealed that RRM2 expression in glioma patients was inversely correlated with the overall survival. Collectively, pectolinarigenin promoted the degradation of CDK1 protein dependent on autolysosomal pathway through increasing autophagic flux by inhibiting RRM2, thereby inhibiting the proliferation of glioblastoma cells by inducing G2/M phase cell cycle arrest, and RRM2 may be a potential therapeutic target and a prognosis and predictive biomarker in GBM patients.

## Introduction

Glioblastoma (World Health Organization grade IV), the most malignant form of intracranial tumor found in adults, accounting for 54% of all gliomas, is incurable due to its high proliferation ability, high invasiveness, resistance to various therapies, and high rate of postoperative recurrence (1). Despite improvements in patients outcomes with the combined radiotherapy and temozolomide regimen since 2005, the median survival duration after the initial diagnosis remains at 15 months with a 5-year survival rate of 7.2% (2). Thus, effective therapeutic approaches for patients with GBM are urgently needed.

Pectolinarigenin (PECT), a dual inhibitor of cyclooxygenase-2/5-lipoxygenase, which is abundantly present in and can be extracted from the Chinese herbal plant *Chromolaena odorata*, has been proven to have numerous pharmacological characteristics, including, anti-inflammatory, anticancer, and anti-allergy activities (3). A previous study also reported that the cancer-specific cytotoxic activity of PECT was mainly attributable to the suppression of proliferative cell cycle, the induction of apoptosis, and the regulation of autophagy (4). However, the benefits of PECT in GBM treatment is not known.

Ribonucleotide reductase subunit M2 (RRM2) encodes the catalytic subunit of ribonucleotide reductase (RNR), which is a hetero-tetramer consisting of two RRM1 and two RRM2 subunits and is involved in regulating DNA synthesis and modifying proteins (5), and is a rate-limiting molecule during the conversion of ribonucleoside triphosphates into dNTPs in the G2 phase of cell cycle (6) and shows elevated levels in some cancers (7, 8). Recent studies demonstrated that overexpression of RRM2 is essential for the cellular response to abnormal dNTP levels, which leads to malignant biological phenotypes such as angiogenesis, epithelial to mesenchymal transition, relapse and drug- or radio-resistance (5, 8). In precision medicine for tumors, targeting of RRM2 has emerged as a therapeutic method for some cancers as it inhibits proliferative cell cycle and regulates autophagy pathways to suppress tumor progress (9). Autophagy is a cellular catabolic process that maintains normal cellular physiological functions by degrading and/or recycling intracellular macromolecules and dysfunctional organelles (10). Moreover, autophagy activation is essential for promoting tumor cell survival and malignant transformation. However, excessive activation or inhibition of autophagy can suppress tumor proliferation and induce cell cycle arrest, and some autophagy genes are involved in cell cycle regulation (11, 12). For example, p62 depletion has been shown to induce cell cycle arrest by promoting cyclin-dependent kinase 1 (CDK1) degradation in human breast cancer (13). In contrast, autophagy related 7 (Atg7) -deficient cells fail to induce p21 expression, thereby impairing p53-mediated cell cycle arrest (14). Although RRM2 is involved in regulating autophagy pathway and cell cycle arrest, it is unclear whether the two biological phenomena are played a role in the anti-tumor effect of PECT on GBM. In this study, we investigate how RRM2 mediates the anti-tumor effect of PECT on GBM *via* reducing CDK1 protein expression and examined the functional significance of autophagy and cell cycle in GBM treatment.

## Materials and Methods

### Patients and Specimens

Normal human brain specimens (n = 6) and fresh glioma specimens (n = 19) were obtained from the Department of Neurosurgery, First Affiliated Hospital of Harbin Medical University. The study protocol was approved by the Clinical Research Ethics Committee of Harbin Medical University. All patients provided written informed consent, and the study was conducted in accordance with the Declaration of Helsinki.

### Cell Lines and Chemical Reagents

The U251, U87, and HUVEC cell lines were provided by the China Infrastructure of Cell Line Resource (National Science and Technology Infrastructure) and cultured in Dulbecco’s Modified Eagle medium (RNBK0465, Sigma-Aldrich, St. Louis, MO, USA) supplemented with 10% fetal bovine serum (AllBio Science, Taichung, Taiwan). MG132, chloroquine (CQ), and PECT were from MedChemExpress (Monmouth Junction, NJ, USA).

### MTT Assay and Clone Formation Assay

The cells were added to a 96-well plate at a density of 5,000 cells/well. At different time points, 10 μL of 3-(4,5-dimethylthiazol-2-yl)-2,5-diphenyl-2H-tetrazolium bromide (MTT; cat# HY-15924, MedChemExpress) dye was added to each well and incubated for 4 h. Finally, we replaced the medium with 150 μL of dimethyl sulfoxide and measured the cell viability (Infinite M200 PRO; Tecan Trading AG, Männedorf, Switzerland). The GBM cells were centrifuged and evenly inoculated into a six-well plate at a density of 800 cells/well. After 14 days of culture at distinct concentrations of PECT, the colonies were measured using the ChemiDoc™ MP imaging software (Bio-Rad Laboratories, Inc., CA, USA).

### Immunofluorescence Analysis

After fixation, permeabilization, and sealing, the treated cells and paraffin-embedded glioma tissue sections were incubated with primary antibody and fluorescent secondary antibody, and images were observed with a fluorescence microscope (Lionheart FX; BioTek, Beijing, China). The following primary antibodies were used: CDK1 (BF0091, 1:500, Affinity, USA), p62 (A19700, 1:100, Abclonal, Wuhan, China), LAMP2 (66301-1-lg, 1:100, Proteintech, Hubei, China), Ki67 (A2094, 1:100, Abclonal, Wuhan, China).

### Cell Transfection

GBM cells were interfered with siRNA, plasmid, and lentiviruses. The siRNA-NC/RRM2 and RRM2 overexpression plasmid pEnter-NC/RRM2 were from Miaolingbio (Wuhan, China) and lentiviruses were from GeneChem (Shanghai, China). The sequences of siRNA-NC/RRM2 and LV-shNC/LV-shRRM2 are shown in **Table 1**.

**Table 1:** Sequences of siRNA, primers, and lentiviruses.

siRNA sequences	
si-NC	5′UUCUCCGAACGUGUCACGUTT3′
RRM2-si RNA1	5′GGCUCAGCUUGGUCGACAA3′, 5′UUGUCGACCAAGCUGAGCC3′
RRM2-si RNA2	5′GGAGAGAGUAAGAGAAAUATT3′
RRM2-si RNA3	5′GAAGAGAGUAGGCGAGUAUTT3′
Primer sequences	
GAPDH	F-5′CACCCACTCCTCCACCTTTGA3′, R-5′ACCACCCTGTTGCTGTAGCCA3′
CDK1	F-5′AAACTACAGGTCAAGTGGTAGCC3′, R-5′TCCTGCATAAGCACATCCTGA3′
RRM2	F-5′TGCCATTGAAACGATGCCTT 3′, R-5′ACTGCAGCAAAGGCTACAAC 3′
Lentivirus sequences	
LV-shNC	5′ TTCTCCGAACGTGTCACGT 3′
LV-shRRM2 1	5′ccCATTTGACTTTATGGAGAA3′
LV-shRRM2 2	5′ gcTCAAGAAACGAGGACTGA3′
LV-shRRM2 3	5′ gcAGATGTATAAGAAGGCAGA3′

### Transmission Electron Microscopy

U251 cells were first fixed with 2.5% glutaraldehyde at 4°C, and post-fixed with 1% osmium tetroxide. The immobilized cells were then dehydrated in increasing concentrations of ethanol and acetone. Finally, Transmission Electron Microscopy (TEM) was used to observe autophagic vesicles.

### Western Blot Analysis 

For Western Blot (WB) analysis, the tissues and treated cells were first extracted and lysed. After separating the proteins using 12.5% sodium dodecyl sulfate-polyacrylamide gel electrophoresis, they were transferred to a polyvinylidene fluoride membrane, which was blocked and then incubated with primary antibodies and fluorescent-dye conjugated secondary antibodies. Protein bands were imaged using a ChemiDoc™ MP Imaging System (Bio-Rad, Hercules, CA, USA). The following primary antibodies were used: RRM2 (BS7520, 1:1,000, BioWorld, Nanjing, China); LAMP2 (66301-1-lg, 1:1,000, Proteintech, Hubei, China); LC3B (83506S, 1:1,000, Cell Signaling Technology, USA); CCNA2 (A7632, 1:1,000), CCNB1 (A2056, 1:1,000), CDK1 (A0220, 1:1,000) and p62 (A19700, 1:1,000) were purchased from Abclonal (Wuhan, China).

### Flow Cytometric Analysis

The treated cells were first digested with 0.25% trysin, centrifuged, and then fixed with 70% ethyl alcohol at 4°C. The next day, the digested cells were stained using a cell cycle analysis kit (P0010, Beyotime, Shanghai, China). Finally, a flow cytometer (Agilent NovoCyte, China) was used to measure the cell cycle distribution.

### RFP-GFP-LC3 Lentivirus Transfection and Fluorescence Imaging

After treatment under different conditions, U251 cells transfected with RFP-GFP-LC3 lentivirus were fixed and used to measure autophagy flux. The fluorescence intensity was analyzed using a FluoView FV300 confocal microscope (Olympus Corporation, Tokyo, Japan).

### Quantitative Real-Time Polymerase Chain Reaction Assay

Total RNA was extracted from the clinical samples or treated cells using TRIzol (Sigma-Aldrich), and then one microgram of total RNA was reverse-transcribed into cDNA using a ReverTra Ace qPCR RT Kit (Toyobo, Osaka, Japan). Quantitative Real-Time Polymerase Chain Reaction (qRT-PCR) was performed on triplicate samples in a reaction mixture of SYBR Green (Roche, Basel, Switzerland) with a Gene Amp PCR System 9700 (Thermo Fisher Scientific, Waltham, MA, USA). The data were normalized to glyceraldehyde 3-phosphate dehydrogenase (GAPDH) using the ΔΔCt method. The primer sequences for GAPDH, RRM2 and CDK1 are shown in **Table 1**.

### Co-Immunoprecipitation

First, the primary antibody was added to Protein A/G Plus-Agarose and incubated at 4°C for 2 h, and then the cell protein lysates were added and incubated at 4°C overnight. We next separated the magnetic beads, collected the supernatant, and performed WB.

### Liquid Chromatography-Mass Spectrometry

Each tumor bearing mice brain tissue sample was mixed with methanol (containing 5 μg/mL 2-chloro-L-phenylalanine as an internal standard) and mixed with a vortex mixer for homogenization. The samples were centrifuged and transferred to sampler vial. An in-house quality control (QC) sample was prepared by mixing equal amounts of each sample. We performed Liquid Chromatography-Mass Spectrometry (LC-MS) analysis of the QC samples. The raw data was converted to a common format, and the degree of aggregation of QC samples was assessed using the principal component analysis modeling method and PCA modeling method was used to check the aggregation degree of QC simples.

### *In Vivo* Studies

BALB/c nude mice were purchased from Beijing Vital River Laboratory Animal Technology Co., Ltd. (Beijing, China). For each mouse, 5 × 10^6^ U251 cells were subcutaneously implanted into the right flank, or 1 × 10^6^ U251 cells were implanted into the brain. The mice were randomly divided into control and PECT groups (six mices per group). PECT (25mg/kg every other day, prepared as a 150 mM stock solution in DMSO and stored at ‐20°C) diluted with 100 μL PBS was administered intraperitoneally for 3 weeks, whereas the control group treated with the same amount of DMSO diluted with 100 μL PBS. Additionally, 1 × 10^6^ U251 cells transfected with LV-shNC or LV-shRRM2 per mice were implanted into the brain. All animal study protocols were approved by the Animal Experiments Ethics Committee of Harbin Medical University and the study was conducted in accordance with the Declaration of Helsinki.

### Hematoxylin and Eosin Staining and Immunohistochemical Staining 

After dehydration and paraffin embedding, the mice brain tumor and organ sections were stained with Hematoxylin and Eosin (H&E). For Immunohistochemical (IHC) assay, tumors were first formalin-fixed, paraffin-embedded, and sliced, and then the sample sections were immunostained with primary antibodies and an anti-mouse/rabbit secondary antibody.

### RNA-Sequencing and Bioinformatics Analyses

We used DESeq2 or edgeR to analyze differences in expression and identify enriched functional terms and pathways (*via* Gene Ontology [GO] and Kyoto Encyclopedia of Genes and Genomes [KEGG]). The clinical information and mRNA sequencing data of the 1,038 patients with glioma were obtained from the Chinese Glioma Genome Atlas (CGGA) database http://www.cgga.org.cn/index.jsp, which included data from 625 low-grade glioma (LGG), 388 GBM, and 20 non-glioma patients. OS was determined among 592 LGG and 374 GBM cases, and the median was used as the demarcation point.

### Statistical Analysis

Differences between two groups and multiple groups were estimated using Student *t*-test and one-way analysis of variance, respectively. The statistical significance of OS between different groups was evaluated by log-rank test. All statistical analyses were carried using GraphPad Prism version 7.0 software (GraphPad, Inc., San Diego, CA, USA), and a value of *p* < 0.05 was considered to indicate statistically significant results.

## Results

### PECT Suppresses GBM Cells Proliferation *In Vitro* and *In Vivo*

**Figure 1:**
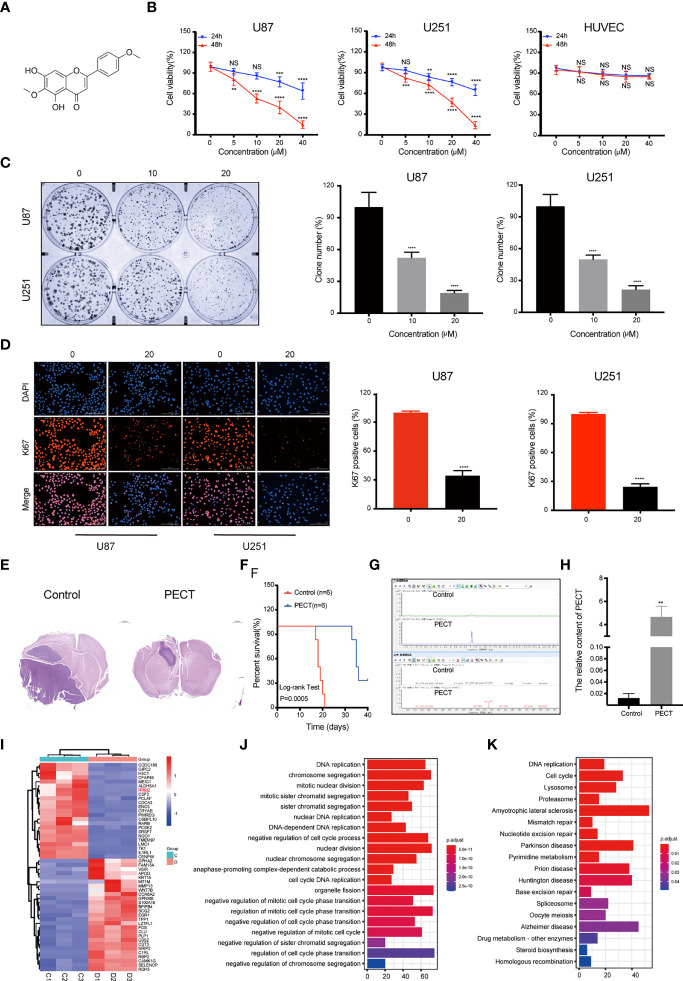
PECT inhibits GBM cells proliferation both *in vitro* and *in vivo*, and RNA-sequencing analysis. **(A)** Structure of PECT. **(B)** After exposuring to different concentrations of PECT for 24 or 48 h, cell viability of U87, U251 and HUVECs cells was assessed using MTT. **(C)** Representative images of colony formation on U87 and 251 cells treated with PECT for different concentrations. **(D)** After U87 and U251 cells were cultured with or without PECT (20 μM, 48 h), Ki67 staining was observed by fluorescence microscopy. Scale bar: 200 μm. **(E)** H&E images of brain sections of mice orthotopically xenografted tumor with U251 cells treated with PECT. Scale bar: 1000 μm. **(F)** Kaplan-Meier survival curve of mice orthotopically xenografted tumor with U251 cells treated with PECT. **(G, H)** LC-MS analysis of PECT content in the tumor bearing mice brain homogenate samples. **(I)** Heatmap of top 50 up- or down-regulated DEGs after PECT treatment. C1-3: control group, D1-3: PECT-treated group. **(J)** GO and **(K)** KEGG analysis of DEGs. The data are presented as the mean ± SD (n=3). NS, non-significant. **P < 0.01, ***P < 0.001, ****P < 0.0001.

The 2D structure of PECT is presented in **Figure 1A**. Firstly, human GBM cells U87 and U251, and human normal cell HUVEC were used to verify the anti-proliferation effect of PECT. MTT results showed that PECT inhibited GBM cells proliferation in dose- and time-dependent manners, but not that of HUVECs. The half-maximal inhibitory concentration (IC_50_) values of PECT for 48 h among U87 and U251 cells were 21.17 and 21.00 μM, respectively (**Figure 1B**). Therefore, the drug concentration closest to IC_50_ of 20 μM was selected for follow-up experiments. Subsequently, we used clonogenic assays to verify the sensitivity of GBM cells to PECT and founded that PECT can prevent the tumor sphere formation in a dose-dependent manner (**Figure 1C**). Additionally, PECT inhibited GBM cells proliferation as shown using ki67 staining (**Figure 1D**). Therefore, PECT inhibited GBM cells proliferation and can be administered safely *in vitro*.

The mice accepted the treatment of PECT showed a much smaller brain tumor size than another (**Figure 1E**) and had a significantly prolonged lifespan (**Figure 1F**). In addition, we performed LC-MS assays to detect the concentration of PECT in gliomas and found that PECT can penetrate the blood-brain barrier (BBB) (**Figures 1G, H**, **Figure S1**), which was consistent with the outcomes described in the traditional Chinese medicine systems pharmacology database and analysis platform (TCMSP) (http://lsp.nwu.edu.cn/tcmsp.php) (**Table S1**) (15). These data indicate that PECT had an anti-tumor effect on GBM *in vivo*.

### PECT Downregulates RRM2 Expression and Increased Autophagy Flux *In Vitro*

**Figure 2:**
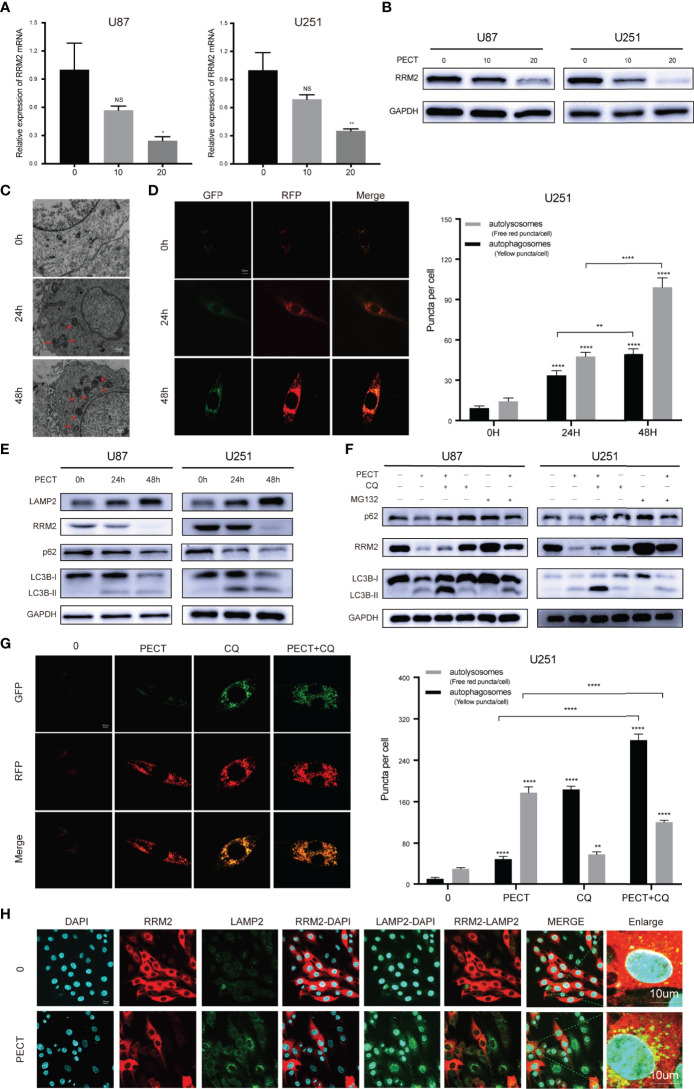
PECT downregulates RRM2 expression and increases autophagy flux *in vitro*. **(A, B)** After culturing U87 and U251 cells with different concentrations of PECT for 48h, RRM2 mRNA and protein expression were examined by qRT-PCR and western blot assay, respectively. **(C)** TEM images of U251 cells exposed to PECT (20 μM) for 0h, 24h, and 48 h. Autophagic vacuole (red arrows). Scale bar: 10 μm. **(D)** After U251 cells cultured with PECT (20 μM) for 0h, 24h, and 48 h, autophagic flux was analyzed using the RFP-GFP-LC3 construct. Scale bar: 20 μm. **(E)** Western blot analysis of RRM2, p62, LC3B and LAMP2 protein expression in U87 and U251 cells cultured with PECT (20 μM) for 0h, 24h, and 48 h. **(F)** Western blot analysis of RRM2, p62 and LC3B protein expression in U87 and U251 cells cultured with PECT (20 μM, 48 h) or CQ (2 μM, 2 h) or MG132 (2 μM, 3 h). **(G)** After U251 cells cultured with PECT (20 μM, 48h) or CQ (2 μM, 2h), autophagic flux was analyzed using the RFP-GFP-LC3 construct. Scale bar: 20 μm. **(H)** Immunofluorescence analysis of RRM2 (red) and LAMP2 (green) in U251 cells treated with or without PECT(20 μM, 48 h). The nuclei were stained with DAPI. Scale bar: 10 μm. The data are presented as the mean ± SD (n=3). NS, non-significant. *P < 0.05, **P < 0.01, ****P < 0.0001.

To determine the mechanism of the anti-tumor effect of PECT on GBM, we screened differentially expressed genes (DEGs) using RNA-Sequencing (RNA-Seq) analysis after PECT treatment, and detected 1,279 DEGs (662 upregulated genes and 617 downregulated genes, |log2 fold-change|>1.0, *p*<0.05), the top 50 of which are shown in **Figure 1I**. The functional annotation analysis of the DEGs using GO. DNA replication and cell cycle regulation were the mainly GO terms (p.adjust <0.05) (**Figure 1J**). In addition, KEGG enrichment pathway (*p*.adjust <0.05) analysis revealed that the DNA replication, cell cycle, lysosomal, and proteasome pathways were the major signs pathways influenced by PECT (**Figure 1K**). Among the top 50 of DEGs, RRM2 was closely correlated with DNA replication, cell cycle, lysosomal and proteasome pathways, so we hypothesized that RRM2 may be a key target gene for the anti-tumor effect of PECT on GBM. To verify the changes of RRM2 expression, we exposed GBM cells to PECT for 48 h and founded that RRM2 mRNA and protein expression gradually decreased with increasing PECT concentrations (**Figures 2A, B**).

Next, we investigated whether PECT regulates autophagy in GBM cells. TEM results revealed that autophagic vacuoles gradually increased after adding PECT to U251 cells from 0 h to 48 h (**Figure 2C**); simultaneously, LC3B-II and LAMP2 protein expression increased and p62 protein expression decreased (**Figure 2E**), indicating that PECT increased autophagic vacuoles in GBM cells by increasing autophagic flux. Further, we performed a RFP-GFP-LC3 transfection assay on U251 cells to re-confirm the above results, and found that both free red puncta (autolysosomes) and yellow puncta (autophagosomes) increased in the merged sections of U251 cells treated with PECT in a time-dependent manner, and the red puncta increased more (**Figure 2D**), validating the increase of autophagic flux.

Subsequently, we investigated whether the autolysosome was involved in RRM2 degradation. chloroquine (CQ), a late-stage autophagy inhibitor, of which low dose that can partially inhibit pH value increase and block protein degradation in the autolysosome, but has no effect on cell activity (16), was used. The results showed that 2 μM CQ did not affect the cell viability of GBM (**Figure S2**), but increased p62 and LC3B-II protein expression, indicating inhibition of autophagic flux (**Figure 2F**). Therefore, 2 μM CQ was selected. An increased number of yellow puncta but a decreased number of free red puncta was observed in the merged sections of the PECT+CQ-treated groups compared to the PECT-treated groups (**Figure 2G**); simultaneously, the reduction of the p62 and RRM2 protein expression was partially recovered, but the LC3B-II protein expression further increased (**Figure 2F**), indicating that CQ blocked the autophagic flux induced by PECT and inhibited RRM2 protein degradation. As expected, immunofluorescence assay of LAMP2 [a lysosomal membrane marker that is involved in autophagy and critical for some proteins degradation in lysosomes (17)] and RRM2 showed that PECT treatment resulted in more LAMP2 protein expression but less RRM2 protein expression in U251 cells, and increased the cytoplasmic co-localization of LAMP2 and RRM2 (**Figure 2H**), further indicating that RRM2 depends on autolysosome pathway degradation.

Additionally, we verified that the proteasome is also involed in RRM2 protein degradation, which can be partly reversed by the proteasome inhibitor MG132 (**Figure 2F**).

Overall, our *in vitro* data confirmed that PECT treatment can increase autophagy flux in GBM cells, and RRM2 protein reduction after PECT treatment is not only dependent on transcriptional inhibition but also proteasomal and autolysosomal degradation.

### RRM2 Knockdown Inhibits GBM Proliferation and Induces Cell Cycle Arrest *In Vitro* and *In Vivo*

**Figure 3:**
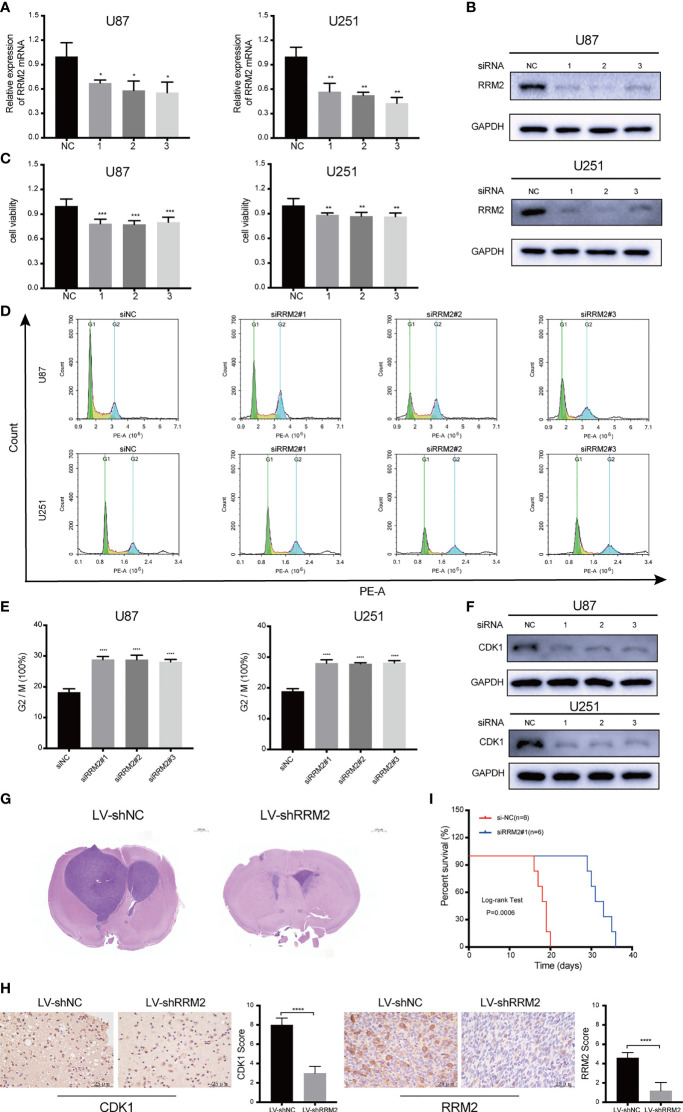
RRM2 knockdown inhibits GBM proliferation and induces cell cycle arrest both *in vitro* and *in vivo*. After transfecting U87 and U251 cells with RRM2 siRNA for 48h, **(A)** RRM2 mRNA expression was examined by qRT-PCR, **(B)** RRM2 protein expression was analyzed by western blot assay, **(C)** cells viability were assessed using MTT, **(D, E)** cells cycle distribution were analyzed using flow cytometry. (green: G0-G1, yellow: S, and blue: G2-M). **(F)** CDK1 protein expression was analyzed by western blot assay. **(G)** H&E images of brain sections of mice orthotopically xenografted with U251 cells transfected with LV-shNC and LV-shRRM2. Scale bar: 1000 μm. **(H)** IHC staining of CDK1 and RRM2 in consecutive brain sections of mice orthotopically xenografted with U251 cells transfected with LV-shNC and LV-shRRM2. Scale bar: 25 μm. **(I)** The survival time of nude mice orthotopically xenografted with U251 cells transfected with LV-shNC and LV-shRRM2. The data are presented as the mean ± SD (n=3). *P < 0.05, **P < 0.01, ***P < 0.001, ****P < 0.0001.

To assess the effect of RRM2 on GBM, we used siRNA to interfere with RRM2 expression in GBM cells. The siRNA efficiency was shown in **Figure 3A**; similarly, RRM2 knockdown not only decreased RRM2 protein expression (**Figure 3B**) but also suppressed GBM cells viability (**Figure 3C**). Moreover, we founded that the proportion of G2/M phase cells increased (**Figures 3D, E**) and CDK1 protein expression was decrease with RRM2 knockdown (**Figure 3F**), but CCNA2 and CCNB1 protein expression no change (**Figure S3**). In addition, we founded that the expression of CDK1 mRNA was also not change (**Figure S4**). These results demonstrated that G2/M cell cycle arrest is caused by the decrease of CDK1 protein, which is consistent with previous reports (18, 19).

Subsequently, RRM2 knockdown was induced by RRM2 lentivirus in U251 cells. After testing the transfection efficiency (**Figure S5**), lentivirus-treated cells were implanted into the brains of 5-week-old nude mice. Mice treated with RRM2 lentivirus showed much smaller tumor volumes compared to controls (**Figure 3G**) and exhibited significantly prolonged survival time (**Figure 3I**). IHC analysis showed that compared with the LV-shNC group, the protein expression of CDK1 and RRM2 decreased in LV-shRRM2 group (**Figure 3H**).

Taken together, our results revealed that RRM2 knockdown could induce G2/M phase cell cycle arrest by reducing the level of CDK1 protein and inhibit GBM cells proliferation *in vitro* and *in vivo*.

### RRM2 Knockdown Induces Cell Cycle Arrest in GBM *via* Promoting CDK1 Protein Degradation by Increasing Autophagic Flux *In Vitro*

**Figure 4:**
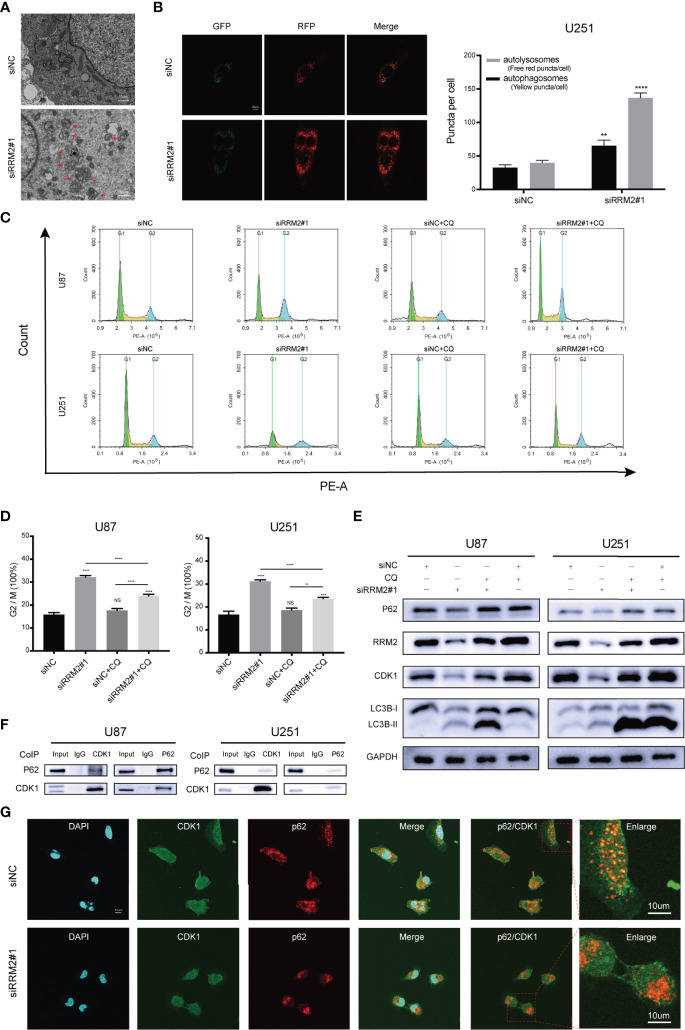
RRM2 knockdown induces cell cycle arrest in GBM *via* promoting CDK1 protein degradation by increasing autophagic flux *in vitro*. **(A)** TEM images of U251 cells transfected with RRM2 siRNA for 48h. Autophagic vacuole (red arrows). Scale bar: 10 μm. **(B)** U251 cells transfected with RRM2 siRNA for 48h, autophagic flux was analyzed using the RFP-GFP-LC3 construct. Scale bar: 20 μm. **(C, D)** After U87 and U251 cells transfected with RRM2 siRNA for 48 h or cultured with CQ (2 μM, 2 h), cells cycle distribution were analyzed using flow cytometry. (green: G0-G1, yellow: S, and blue: G2-M). **(E)** After U87 and U251 cells transfected with RRM2 siRNA for 48 h or cultured with CQ (2 μM, 2 h), CDK1, RRM2, p62 and LC3B proteins expression were analyzed by western blot assay. **(F)** Total protein lysates of U87 or U251 cells were prepared for Co-IP using CDK1 or p62 antibody. **(G)** Immunofluorescence assay of the co-localization of p62 (red) and CDK1 (green) in U251 cells after transfection with RRM2 siRNA for 48 h. Scale bar: 10 μm. The data are presented as the mean ± SD (n=3). NS, non-significant. **P < 0.01, ***P < 0.001, ****P < 0.0001.

Firstly, we founded that RRM2 knockdown can promote the autophagic vacuoles accumulated in U251 cells (**Figure 4A**). Simultaneously, a RFP-GFP-LC3 transfection assay revealed that both free red puncta and yellow puncta increased in the merged sections of si-RRM2#1 U251 cells compared with that in si-NC U251 cells, but the free red puncta increased more (**Figure 4B**), indicating the autophagic flux increased.

Subsequently, we sought to determine whether autophagy is required for RRM2-mediated cell cycle progression. The data showed that RRM2 knockdown promoted G2/M phase cell cycle arrest, while this effect was reversed by CQ, an inhibitor of autophagic flux that prevents autophagosome-lysosome fusion and lysosomal protein degradation (**Figures 4C, D**). Interestingly, the change in CDK1 protein level was consistent with the cell cycle distribution (**Figure 4E**). These data indicate that the blockage of G2/M phase cell cycle induced by RRM2 knockdown is dependent on the function of RRM2 in autophagy.

To further investigate the mechanism regarding whether the degradation of CDK1 protein depends on the autolysosomal pathway during RRM2 knockdown, we performed Co-Immunoprecipitation (Co-IP) assays to analyze the interaction between CDK1 and p62, an adapter protein, is required for some protein degradation through the autolysosome pathway (20). As shown in **Figure 4F**, p62 was able to interact with CDK1. Additionally, immunofluorescence analysis showed that CDK1 could co-localize with p62 in U251 cell, but the co-localization of p62 with CDK1 decreased after RRM2 knockdown (**Figure 4G**). Thus, our results demonstrated that RRM2 knockdown induced cell cycle arrest in GBM *via* promoting CDK1 protein degradation by increasing autophagic flux *in vitro*.

### PECT Inhibits GBM Proliferation, Promotes Cell Cycle Arrest, and Increases Autophagic Flux by Decreasing RRM2 Expression *In Vitro*

**Figure 5:**
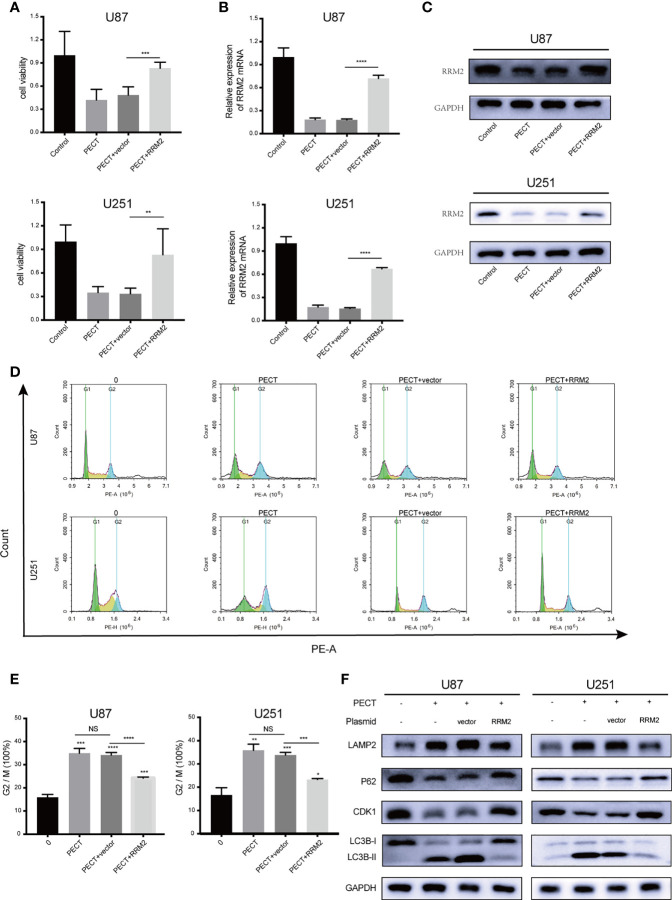
PECT inhibits GBM proliferation, promotes cell cycle arrest, and increases autophagic flux by decreasing RRM2 expression *in vitro*. After U87 and U251 cells transfected with RRM2 overexpression plasmid or cultured with PECT (20 μM, 48 h), **(A)** cells viability were assessed using MTT, **(B)** RRM2 mRNA expression was examined by qRT-PCR, **(C)** RRM2 protein expression was analyzed by western blot assay, **(D, E)** cells cycle distribution were analyzed using flow cytometry. (green: G0-G1, yellow: S, and blue: G2-M). **(F)** CDK1, p62, LAMP2 and LC3B protein expression were analyzed by western blot assay. Vector and RRM2 represent negative control plasmid and RRM2 overexpression plasmid, respectively. The data are presented as the mean ± SD (n=3). NS: non-significant. *P < 0.05, **P < 0.01, ***P < 0.001, ****P < 0.0001.

To verify whether RRM2 mediates the anti-tumor effect of PECT on GBM, we overexpressed RRM2 and exposed them to PECT, and founded that GBM cells viability (**Figure 5A**) were partially rescued with RRM2 expression upregulation (**Figures 5B, C**). Similarly, the increased proportion of G2/M phase GBM cells (**Figures 5D, E**) and the downregulation of p62 and CDK1 protein and upregulation of LAMP2 and LC3B-II protein caused by PECT were partially recovered with RRM2 expression upregulation (**Figure 5F**). Overall, our results showd that PECT can inhibit GBM proliferation and promote G2/M phase cell cycle arrest as well as increase autophagic flux by decreasing RRM2 expression *in vitro*.

### PECT Inhibits GBM Proliferation *In Vivo*

**Figure 6:**
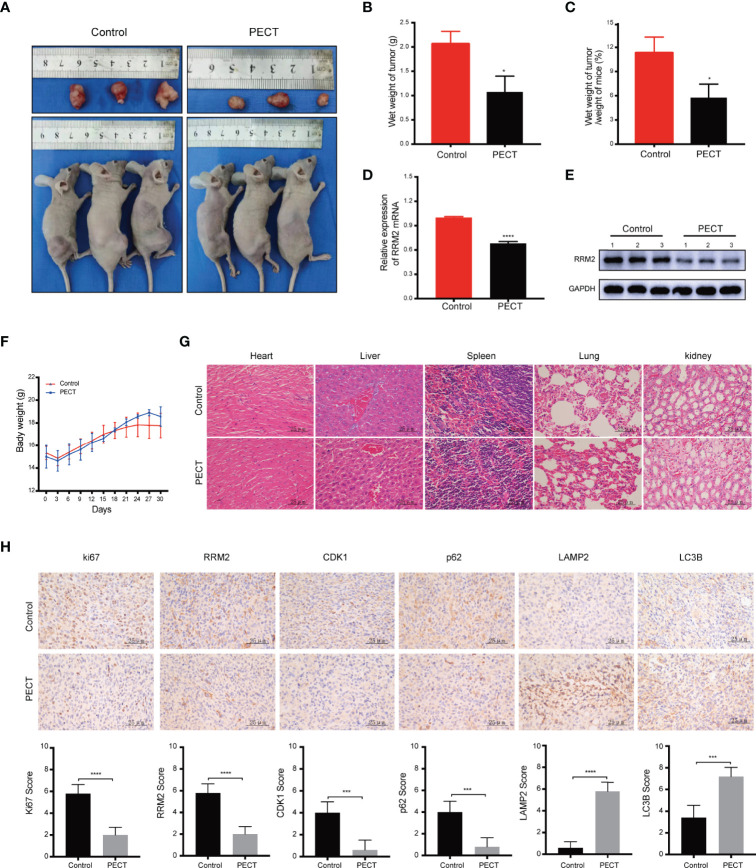
PECT inhibits GBM proliferation *in vivo*. **(A)** Dissected images of mices and tumors, **(B)** tumor wet weight and **(C)** wet weight of tumor/weight of mice between the control and PECT groups. **(D, E)** RRM2 mRNA and protein expressions in mouse tumors with or without PECT treatment. **(F)** Body weight changes in the mouse models. **(G)** Representative images of H&E staining of the organs from mice with or without PECT treatment. Scale bar: 25 μm. **(H)** IHC staining for Ki67, RRM2, CDK1, p62, LAMP2, and LC3B in xenograft tumors between the control and PECT groups. Scale bar: 25 μm. The data are presented as the mean ± SD (n=3). *P < 0.05, ***P < 0.001, ****P < 0.0001.

*In vivo*, our results showed that PECT suppressed tumor proliferation, accompanied by reducing tumor weight (**Figures 6A, B**) and tumor weight/mouse weight (**Figure 6C**), and downregulating RRM2 mRNA (**Figure 6D**) and protein (**Figure 6E**) expression compared to that in controls. However, the body weight of mice in each group did not differ significantly (**Figure 6F**). Moreover, we founded that PECT itself did not induce additional toxicities by evaluating morphological changes in tissues in all groups (**Figure 6G**). IHC results showed that compared with the control group, the protein expression of LC3B and LAMP2 increased while that of Ki67, RRM2, p62, and CDK1 decreased in xenograft tumors administered with PECT (**Figure 6H**). Taken together, PECT exerts an anti-tumor effect in GBM and can be safely administered *in vivo*.

### RRM2 Is Increased in GBM Tissues and Inversely Correlated With the Prognosis of Glioma Patients

**Figure 7:**
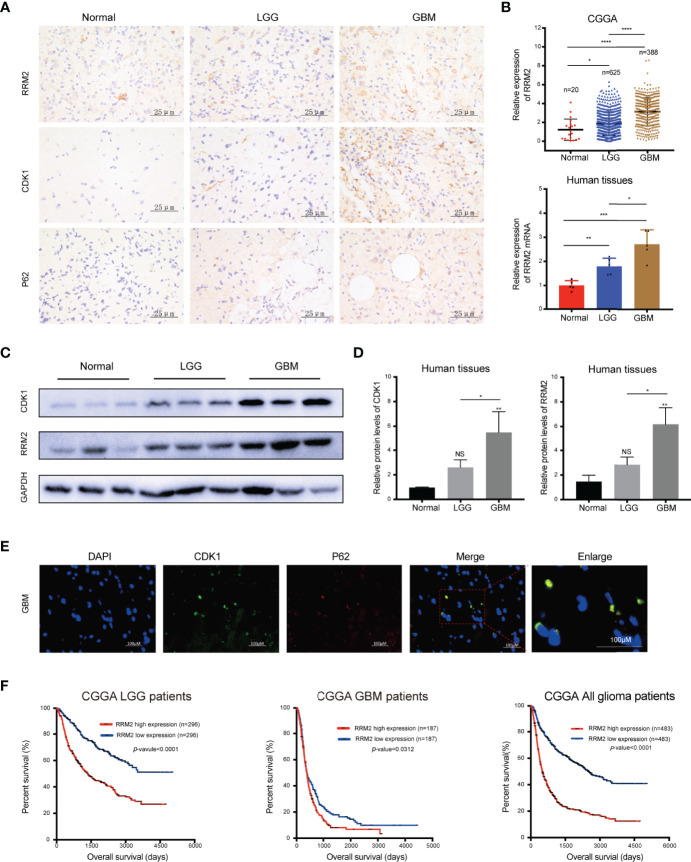
RRM2 is increased in GBM tissues and inversely correlated with the prognosis of glioma patients. **(A)** IHC analysis of RRM2, CDK1, and p62 in sections obtained from primary glioma sample tissues and normal human brain sample tissues. **(B)** Analysis of RRM2 mRNA expression in GBM samples compared to normal human brain samples and LGG samples according to the CGGA data and qRT-PCR results. **(C, D)** WB analysis of RRM2 and CDK1 in GBM sample tissues compared to normal human brain sample tissues and LGG sample tissues. **(E)** Immunofluorescence double-staining analysis showing co-localization of p62 and CDK1 in the same lesional cells in human GBM cases. **(F)** OS curves of patients with glioma according to the median of RRM2. The data are presented as the mean ± SD (n=3). NS, non-significant. *P < 0.05, **P < 0.01, ***P < 0.001, ****P < 0.0001.

To verify the clinical significance of RRM2 in glioma patients, firstly, we performed IHC staining analysis, and founded that compared with normal human brain tissues, RRM2, CDK1, and p62 protein expression had increase in distinct grades of glioma, especially in GBM tissues (**Figure 7A**). Additionally, according to the CGGA data and qRT-PCR results, compared with non-tumor brain tissues, RRM2 mRNA expressions gradually increased with increasing the degree of glioma malignancy (**Figure 7B**). WB analysis revealed that RRM2 and CDK1 protein expression in human GBM tissue was higher than that in LGG and normal human brain tissues, but no significant difference between LGG and normal human brain tissues, indicating that RRM2 may be a predictive marker of GBM (**Figures 7C, D**).

We confirmed that p62 can directly interact with CDK1 and is an adapter for the degradation of CDK1 protein in autolysosome *in vitro*. Therefore, we performed immunofluorescence staining, and founded that p62 and CDK1 protein are co-located in the lesional cells from human GBM cases, which indicated that p62 and CDK1 protein could interact directly *in vivo* (**Figure 7E**). More importantly, we examined the clinical data of patients with glioma in the CGGA database and revealed that RRM2 expression was inversely correlated with the OS time, indicating that RRM2 may be a prognosis marker of GBM (**Figure 7F**).

## Discussion

Chemotherapy is one of the conventional treatments for GBM. However, the existing chemotherapeutic drugs are insufficient to provide a survival period of more than 15 months to GBM patients (21, 22). Nearly a third of the drugs used in clinical care, currently, to treat cancer, come from natural products or its derivatives (23), indicating an attractive prospect for the development of natural products as a novel GBM therapy. PECT, a natural flavonoid, has demonstrated several anticancer activities, including autophagy and G2/M phase cell cycle arrest induction, cell proliferation, and migration inhibition (3, 24, 25). In the present study, we demonstrated for the first time that PECT can suppress GBM proliferation both *in vitro* and *in vivo*. More importantly, PECT significantly suppressed GBM cells, but not HUVECs, proliferation and did not caused morphological changes in organ tissues, indicating that PECT can inhibit tumor growth and can be safely administered *in vitro* and *in vivo*. Effective drugs for treating GBM are currently limited; one of the major limitations to drug development is that therapeutic agents must possess the ability to cross BBB (26). Our results revealed that PECT can not only cross BBB, but also suppress the growth of intracranial GBM in nude mice and prolonged their effective survival time. However, the underlying molecular mechanism of the antineoplastic effect of PECT on GBM remains unclear.

We performed RNA-Seq analysis and found PECT treatment could inhibit the expression of RRM2 in U251 cells and affect the signal pathways such as DNA replication, cell cycle, lysosomal, and proteasome. In addition, qRT-PCR and western blotting assays further verified that PECT could reduce the expression of RRM2 mRNA and protein in GBM cells. RRM2 is the catalytic subunit of heterodimeric tetramer RNR and catalyzes *de novo* formation of dNTPs (27). The level of RRM2 changes continuously during the cell cycle to maintain the balance between dNTP production and DNA synthesis. Multiple cancers, such as melanoma, colorectal cancer, prostate cancer, liver cancer, breast cancer, glioma, and ovarian cancer, benefit from therapy targetting RRM2 (5, 28, 29). Sun and coworkers have shown that silencing RRM2 can induce G2/M phase cell cycle arrest of U87 cells (30). Chen et al. reported that RRM2 knockdown can reverse the resistance of human lung squamous carcinoma cells to gemcitabine by inducing autophagy (31). Our results showed that PECT can induce G2/M phase cell cycle arrest and increase autophagic flux of glioblastoma cells by inhibting RRM2 expression, which can be reversed by RRM2 overexpression plasmid.

Additionally, we found that PECT could inhibit RRM2 transcription and promote RRM2 protein degradation through a proteasome-dependent pathway, consistent with previous report. Such as RRM2 is ubiquitinated by APC/CCDH1 or cyclin F, and then degraded through the proteasome-dependent pathway (32, 33). Transcription factors, including Sp1, AP-2, BRCA, E2F1 and MYBL2, bind to the DNA sequence in the promoter region of *RRM2* and regulate their expression in response to DNA damage in cancer cells (34, 35). We did not thoroughly explore the effect of PECT on RRM2 transcription suppression and proteasome degradation, which will be verified in furture studies. However, we found that in addition to transcriptional suppression and proteasome-dependent degradation, RRM2 protein also depend on autophagy-lysosome pathway degradation. It was reported that excessive activation of autophagy could result in type II programmed cell death, which differs from necrosis and apoptosis (36). Additionally, the dual role of autophagy in response to anticancer therapy is well-known. Although there was some controversy regarding whether autophagy should be turned on or off to treat cancer (12), at least in this context, our data confirmed that RRM2 downregulation mediates the anti-proliferation effect of PECT on GBM, which depended on the increase of autophagic flux. Because PECT inhibited RRM2 expression, which can be inhibited by autophagy inhibitor CQ. In addition, immunofluorescence assay showed that RRM2 can co-localize with LAMP2 in U251 cells, while PECT treatment can increase the intracellular co-localization of LAMP2 and RRM2, and decrease the RRM2 protein expression. Thus, these further demonstrated that PECT could promote RRM2 protein degradation by autolysosome-dependent pathway in GBM.

In proliferating cells, RRM2 overexpression is regulated in a G2/M phase cell cycle-dependent manner (37, 38). Additionally, RRM2 downregulation has been demonstrated to promote autophagy-dependent cell death by decreasing intracellular dNTP levels (31). Reduction in RNR activity could suppress DNA replication and damage repair, resulting in cell cycle arrest (9, 39), and Autophagy has played a role in various cellular processes like DNA damage repair and cell cycle regulation (11, 40, 41). However, the effect of autophagy as a multifunctional regulator on cell cycle progression in GBM remains unclear. In the present study, we confirmed that RRM2 knockdown can induce G2/M phase cell cycle arreste by decreasing CDK1 protein expression, which can be reversed by the inhibition of autophagy. CDK1, an essential kinase regulating cell cycle progression, is upregulated in multiple cancers. CDK1 depletion promote cell-cycle arrest and ultimately inhibit tumor cell proliferation (42–44). Additionally, p62 is an adapter that is widely involved in protein interactions. Our results demonstrated that CDK1 can interact with p62, which provided a basis for autophagy to degrade CDK1. Simultaneously, immunofluorescence assay showed that CDK1 can co-localize with p62 in U251 cells, and RRM2 knockdown can decrease the intracellular co-localization of CDK1 and p62, and decrease the CDK1 protein expression. These data indicated that autophagy is required for RRM2 to inhibit GBM cell proliferation through inducing G2/M phase cell cycle arrest by promoting CDK1 protein degradation, which supported by previous studies that p62-HDAC6 promoted CDK1 degradation through an autophagy-lysosome pathway in breast cancer (13). However, the exact binding site at which CDK1 binds p62 was not identified.

Clinical data revealed that RRM2 is increased in GBM tissues and inversely correlated with glioma sufferers OS time, which suggests RRM2 as a potential therapeutic target, as well as a prognosis and predictive biomarker. Additionally, we found that p62 and CDK1 protein were co-located in the lesional cells from human GBM cases, indicating that autophagy and cell cycle may be related to the occurrence and development of GBM, which may play a certain role for GBM clinical research. We also demonstrated that PECT or RRM2 knockdown was involved in regulating GBM cells migration, and PECT could directly bind to RRM2, and promote CDK1 protein degradation through proteasomal pathway, although the corresponding molecular mechanisms remain to be determined.

## Conclusions

**Figure 8:**
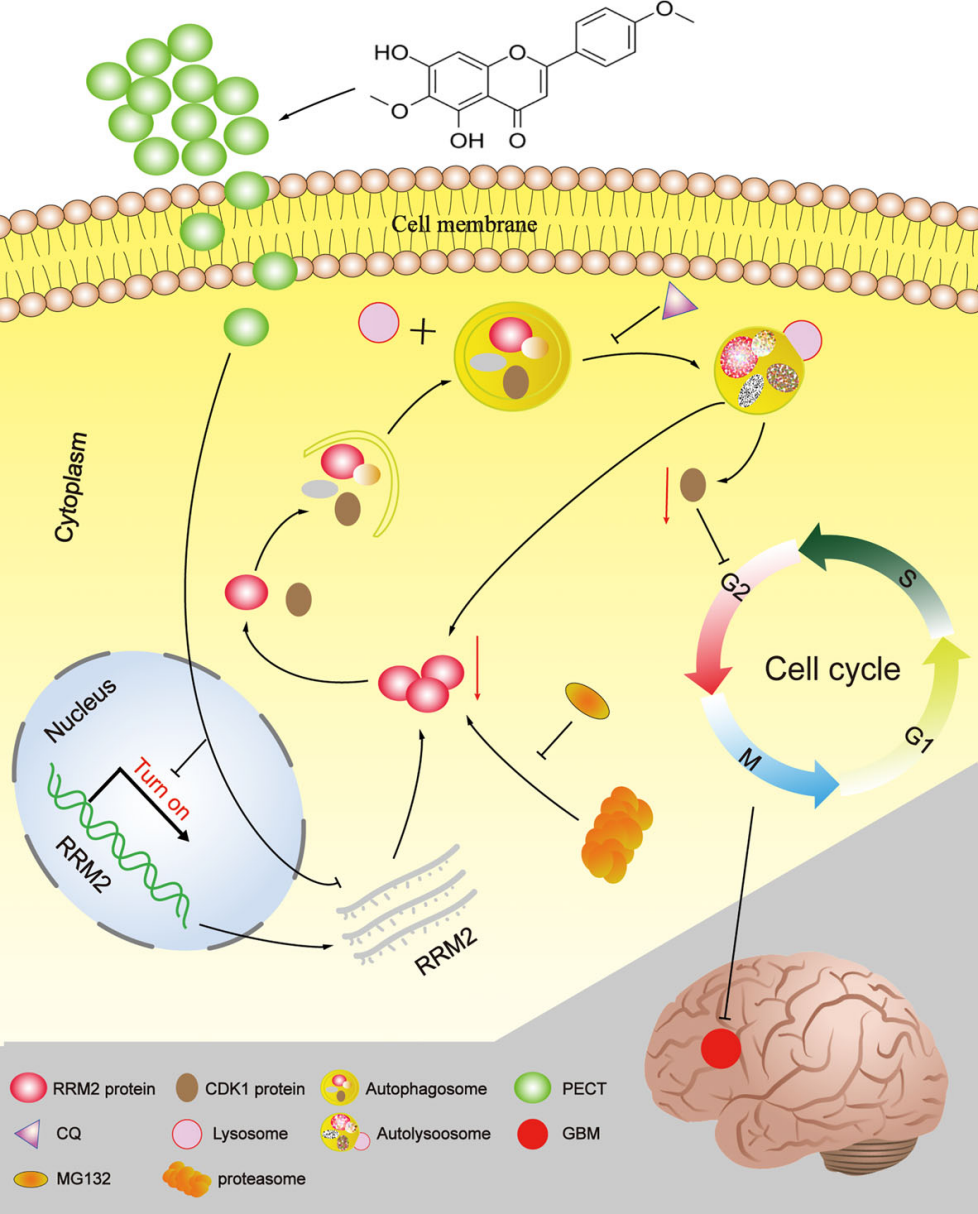
The schematic diagram of RRM2 mediates the anti-tumor effect of PECT on GBM.

In summary, Our study provides a novel insight into the mechanisms by which PECT promoted the degradation of CDK1 protein dependent on autolysosomal pathway through increasing autophagic flow by inhibiting RRM2 (**Figure 8**). Additionally, RRM2 may be a potential therapeutic target and a prognosis and predictive biomarker in GBM patients, and manipulation of RRM2-mediated autophagy offers promising clinical therapeutic directions for GBM. However, the precise molecular mechanism of RRM2 to regulate autophagy in GBM should be further addressed in the future.

## Data Availability Statement

The original contributions presented in the study are included in the article/**Supplementary Material**. Further inquiries can be directed to the corresponding authors.

## Ethics Statement

The study protocol was approved by the Clinical Research Ethics Committee of Harbin Medical University. All patients provided written informed consent, and the study was conducted in accordance with the Declaration of Helsinki. The patients/participants provided their written informed consent to participate in this study. All animal study protocols were approved by the Animal Experiments Ethics Committee of Harbin Medical University and the study was conducted in accordance with the Declaration of Helsinki.

## Author Contributions

HJ designed, conceived, planned, implemented the experiment, and wrote the original draft. LW, HY and EL assisted in collecting clinical samples. TY assisted in imaging. JY, XW and MG provided experimental guidance, and DZ assisted in completing experiments. KD, WL, CZ, JW, PY, ZS, XR, SA and SS participated in the investigation. XiaoC and ZZ provided financial sponsorship. XinC and SZ supervised and funded the research. All authors contributed to the article and approved the submitted version.

## Funding

This work was funded by the Natural Science Foundation of China grants (81972363), Research Fund for the Postdoctoral Science Foundation of China (2019M661303), and Heilongjiang Province Postdoctoral Fund (LBH-Z19076).

## Conflict of Interest

The authors declare that the research was conducted in the absence of any commercial or financial relationships that could be construed as a potential conflict of interest.

## Publisher’s Note

All claims expressed in this article are solely those of the authors and do not necessarily represent those of their affiliated organizations, or those of the publisher, the editors and the reviewers. Any product that may be evaluated in this article, or claim that may be made by its manufacturer, is not guaranteed or endorsed by the publisher.

## Supplementary Material

Supplementary Figure 1 LC-MS of PECT content using Principal component analysis (PCA).

Supplementary Figure 2 The cytotoxicity effect of chloroquine on U87 and U251 cells analyzed using MTT assays

Supplementary Figure 3 CCNA2 and CCNB1 protein expression no change after RRM2 was silenced.

Supplementary Figure 4 CDK1 mRNA expression no change after RRM2 was silenced.

Supplementary Figure 5 The transfection efficiency of RRM2 lentivirus in U251 cells.
