# Supplementary material for: RRM2 Mediates the Anti-Tumor Effect of the Natural Product Pectolinarigenin on Glioblastoma Through Promoting CDK1 Protein Degradation by Increasing Autophagic Flux
Source: Front Oncol. 2022 May 11;12:887294. doi: 10.3389/fonc.2022.887294 (PMC9150261; doi:10.3389/fonc.2022.887294)
Supplement: Supplementary file 1 [file Table_1.docx]

Table S1. Data regarding pharmacological and molecular properties

| **Name** | **MW** | **AlogP** | **Hdon** | **Hacc** | **OB (%)** | **Caco-2** | **BBB** | **DL** | **FASA-** | **TPSA** | **RBN** | **HL** |
| --- | --- | --- | --- | --- | --- | --- | --- | --- | --- | --- | --- | --- |
| PECT | 314.31 | 2.57 | 2 | 6 | 41.17 | 0.70 | -0.09 | 0.30 | 89.13 | 0.00 | 3 | 16.56 |

Abbreviations: Caco-2, caco-2 permeability; OB, oral bioavailability; Dl, drug likeness; BBB, blood-brain barrier.

Suggested drug screening criteria:

- OB: ≥20%; DL ≥0.1;
- BBB: ＜-0.3 is non-penetrating (BBB-), from -0.3 to +0.3, moderate penetrating (BBB±),and＞0.3 strong penetrating (BBB+);
- HL: Drug half-life ≤4 hours: fast-elimination group, between 4-8 hours are mid-elimination group and ≥8 hours are slow-elimination group;
- TPSA: less than 60 angstroms squared is cell membrane permeable;
- RBN: meets only the criteria of 10 or fewer rotatable bonds for good oral bioavailability.
