# Supplementary figures and images for: RRM2 Mediates the Anti-Tumor Effect of the Natural Product Pectolinarigenin on Glioblastoma Through Promoting CDK1 Protein Degradation by Increasing Autophagic Flux
Source: Front Oncol. 2022 May 11;12:887294. doi: 10.3389/fonc.2022.887294 (PMC9150261; doi:10.3389/fonc.2022.887294)

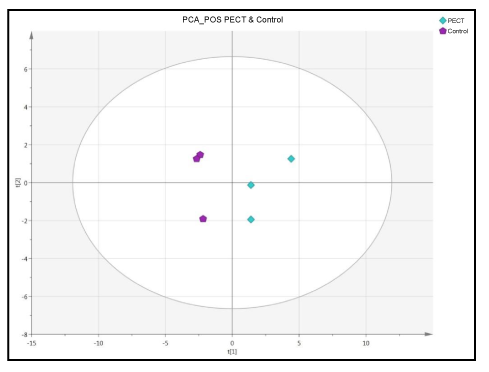

Supplement: Supplementary Figure 1 — LC-MS of PECT content using Principal component analysis (PCA). [file Image_1.tif]

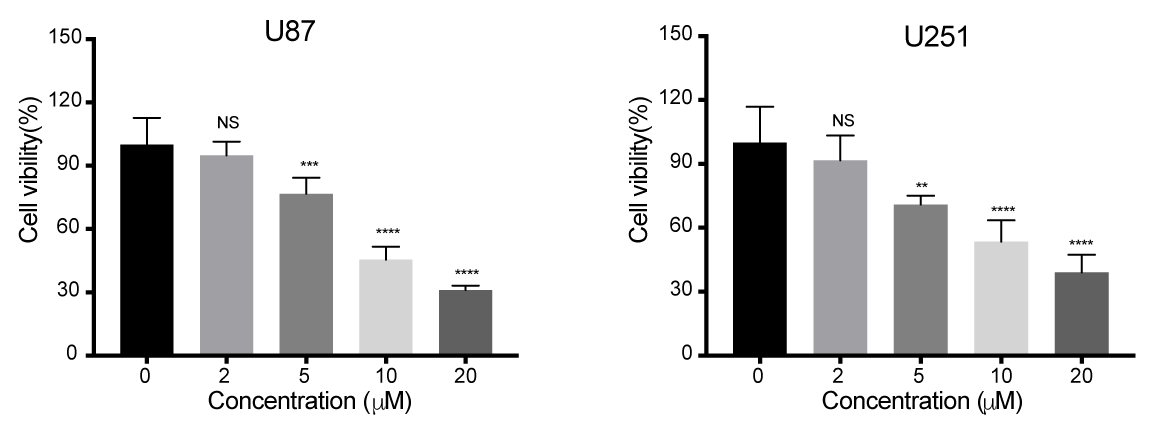

Supplement: Supplementary Figure 2 — The cytotoxicity effect of chloroquine on U87 and U251 cells analyzed using MTT assays [file Image_2.tif]

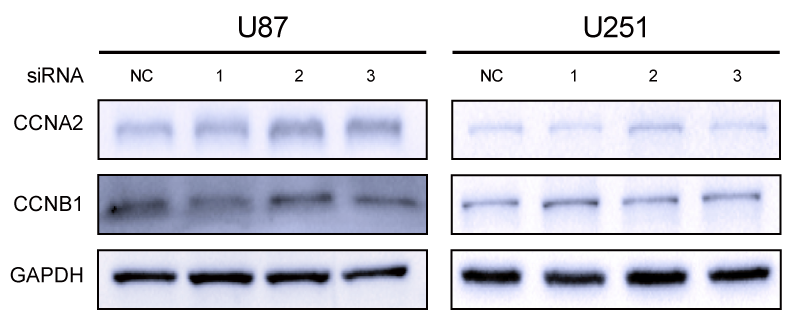

Supplement: Supplementary Figure 3 — CCNA2 and CCNB1 protein expression no change after RRM2 was silenced. [file Image_3.tif]

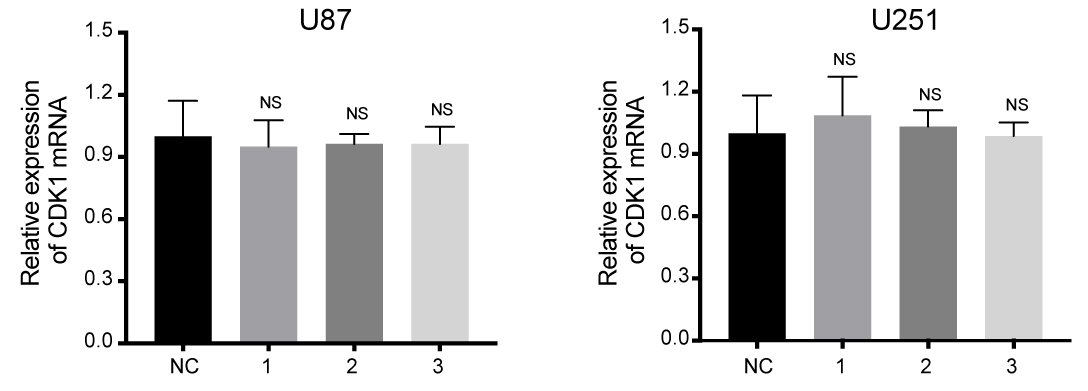

Supplement: Supplementary Figure 4 — CDK1 mRNA expression no change after RRM2 was silenced. [file Image_4.tif]

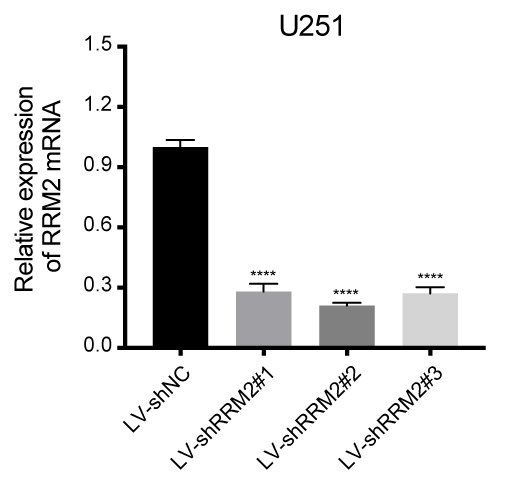

Supplement: Supplementary Figure 5 — The transfection efficiency of RRM2 lentivirus in U251 cells. [file Image_5.tif]
